# Mock Juror Perceptions of Child Witnesses on the Autism Spectrum: The Impact of Providing Diagnostic Labels and Information About Autism

**DOI:** 10.1007/s10803-018-3700-0

**Published:** 2018-07-28

**Authors:** Laura Crane, Rachel Wilcock, Katie L. Maras, Wing Chui, Carmen Marti-Sanchez, Lucy A. Henry

**Affiliations:** 1 Centre for Research in Autism and Education (CRAE), UCL Institute of Education, University College London, London, WC1H 0NU UK; 2 Department of Psychology, University of Winchester, Winchester, UK; 3 Department of Psychology, University of Bath, Bath, UK; 4 Department of Psychology, Goldsmiths, University of London, London, UK; 5 Division of Language and Communication Science, City, University of London, London, UK

**Keywords:** Autism, Criminal justice, Jury, Credibility, Eyewitness memory

## Abstract

Research suggests that autistic children can provide accurate and forensically useful eyewitness evidence. However, members of a jury also rely on non-verbal behaviours when judging the credibility of a witness, and this could determine the verdict of a case. We presented mock jurors with videos (from an experimental study) of one of two child witnesses on the autism spectrum being interviewed about a mock minor crime. Results demonstrated that providing jurors with generic information about autism and/or informing them of the child’s diagnostic label differentially affected credibility ratings, but not for both children. Implications for how to present information about child witnesses with autism to a jury—highlighting the need for approaches tailored to individual children—are discussed.

## Introduction

Eyewitness evidence can be a key factor in a jury’s decision making about a defendant’s guilt or innocence (Nicholson et al. 2014); if jurors do not find a witness to be credible, they are less likely to decide that the defendant is guilty (Pica et al. 2017). In judging the credibility of a witness, jurors consider several factors aside from the content of the witness’ testimony, including expression of emotion (Cooper et al. 2014; Wessel et al. 2013), eye contact (Field et al. 2010), confidence (Dodson and Dobolyi 2015), and surface features of speech (e.g., pause, intonation) (Ozuru and Hirst 2006). Jurors, therefore, rely on non- and para-verbal behaviours, opposed to focusing solely on what the witness has said, when making judgements about their credibility.

As autistic^1^ individuals may be more likely to encounter the criminal justice system than those without autism (e.g., Lindblad and Lainpelto 2011; Turcotte et al. 2017; Woodbury-Smith and Dein 2014), it is crucial to assess how they are perceived in this context (for example, as witnesses). A growing body of empirical evidence has explored the performance of autistic individuals (largely those who do not have intellectual disabilities) when giving evidence in a criminal justice context. This illustrates how both children and adults on the autism spectrum tend to recall less information than their typically developing peers when free recalling information about an event (e.g., Bruck et al. 2007; Henry et al. 2017b; Maras et al. 2012; Mattison et al. 2015; McCrory et al. 2007), but can perform similarly in more structured interviews (e.g., Henry et al. 2017a; Maras and Bowler 2010), or when additional supports (e.g., more specific questioning, physical reinstatement of context, or concrete visual prompts) are provided at recall (e.g., Maras and Bowler 2012a; Maras et al. 2012; Mattison et al. 2016). Research has also highlighted how eyewitness information provided by children with autism can be as accurate as that of comparable peers (Bruck et al. 2007; Henry et al. 2017a, b; McCrory et al. 2007); although it should be noted that accuracy levels can vary with interview type (Mattison et al. 2016) and findings are less consistent in autistic adults (e.g., Maras and Bowler 2010; Maras et al. 2012, 2013). Autistic children and adults may also display atypicalities in narrating memories of personally experienced events; for example, lacking a consistent high point in their narratives (e.g., Goldman 2008; McCabe et al. 2013). Importantly, however, there is no evidence supporting the notion that autistic people are more suggestible than non-autistic people (Bruck et al. 2007; Maras and Bowler 2011, 2012b; McCrory et al. 2007; North et al. 2008). This is despite many legal professionals believing this to be true (see, for example, George et al. 2018, for a survey study on this topic in UK barristers).

Although research suggests that autistic witnesses can give reliable and accurate eyewitness evidence, individuals diagnosed with autism may display atypical behaviours (e.g., unusual eye contact, repetitive body movements) that could result in their credibility being questioned (McCrory et al. 2007). This could be particularly relevant if the person’s autism diagnosis is not disclosed to the jury. Indeed, many witnesses and defendants may be reluctant to share information about their autism diagnosis with criminal justice processionals, for example, due to concerns about justice professionals’ perceived lack of autism of knowledge and awareness (e.g., Crane et al. 2016) or fears that their diagnosis may count against them (e.g., Cooper and Allely 2017). It is, therefore, vital to examine the effects of providing jurors with knowledge of a witness’ autism diagnosis, with or without the provision of additional information about the key characteristics of autism.

Previous research has shown that providing jurors with additional information about witnesses can help address unfair biases held about their credibility. This may be particularly the case for children, who—despite being able to provide accurate and forensically useful eyewitness testimony—often find their credibility questioned (e.g., Lamb et al. 2011). Goodman-Delahunty et al. (2011), for example, found that jurors (in relation to child sexual abuse cases) were more likely to convict a defendant if information that challenged common misconceptions about children’s memory was provided by expert witnesses.^2^ Further, Wadley and Haley (2001) found that providing a diagnostic label (e.g., Alzheimer’s disease, major depression), in addition to information about a person’s condition, allowed others to attribute the person’s atypical behaviour to the diagnosis. Sasson and Morrison (2017) also reported that while first impressions of autistic adults were rated less favourably, informing observers of their autism diagnosis resulted in significantly more favourable judgements about them. Such evidence (that observers can view a person’s behaviours more favourably if they know about a diagnosis) supports Kelley’s (1971, 1972) discounting principle, in which one explanation is diminished when observers are alerted to an alternative one.

In this paper, we present a preliminary study of the perceived credibility of child witnesses on the autism spectrum. This was achieved by presenting mock jurors with an ecologically valid video-recorded interview (as would often be presented to jurors in cases involving child witnesses in England and Wales, as part of the range of Special Measures offered to vulnerable witnesses; YJCEA 1999). These videos were taken from a larger empirical study on the performance of child witnesses—with and without an autism diagnosis—who were interviewed about a staged event that they viewed at their school (see Henry et al. 2017a). Mock jurors rated videos of one of two autistic child witnesses using an 11-item credibility questionnaire, established as useful in previous research on child witnesses with and without intellectual disabilities (Henry et al. 2011; Peled et al. 2004). Prior to completing the questionnaires, we manipulated whether mock jurors were made aware of the children’s diagnoses, to test Kelley’s (1971, 1972) discounting principle. We also manipulated whether providing jurors with additional information about the key characteristics of autism (in addition to the diagnostic label) affected credibility ratings. This is important given that lay people often hold clear, but not necessarily accurate, beliefs about autism (Gillespie-Lynch et al. 2015; Huws and Jones 2010). Following previous research (e.g., Wadley and Haley 2001), it was tentatively predicted that providing information about the child’s autism diagnosis together with information about autism may improve mock juror’s ratings of the child witnesses’ credibility. However, given the heterogeneity found across the autism spectrum, it was also predicted that this may be different for each child witness.

## Method

### Design

We employed a between-subjects design, with mock jurors quasi-randomly assigned (based on age and gender) to view one of two videos (‘Child A’ or ‘Child B’) and to one of three experimental conditions: ‘Told AUT + info’, in which mock jurors were told that the child in the video had an autism diagnosis and were provided with information about the key characteristics of autism (see “Appendix”); ‘Told AUT + no info’, in which mock jurors were told that the child had an autism diagnosis but were not provided with further information about autism; or ‘Not told AUT + no info’, in which mock jurors were not told that the child had an autism diagnosis, nor were they given any information about autism. The dependent variables were 11 credibility characteristics on which the mock jurors rated the child (from Henry et al. 2011; Peled et al. 2004).

### Participants

An opportunity sample of 120 mock jurors participated in this study, all of whom confirmed their eligibility for jury service in the United Kingdom: the jurors were aged 18–69, were not lacking capacity within the meaning of the Mental Capacity Act (Department of Health 2005), and were not recently serving criminal convictions. Half of the jurors (n = 60) watched Child A’s video, and the other half (n = 60) watched Child B’s video. Within each group, 20 jurors were assigned to the ‘Told AUT + info’ condition, 20 were assigned to the ‘Told AUT + no info’ condition, and 20 were assigned to the ‘Not told AUT + no info’ condition. Mock juror demographics are presented in Table 1, along with ratings of their prior knowledge/experience of autism.

**Table 1:** Mock juror demographics (n = 120)

Video	Condition	N	Mean age (SD)	Gender (M:F)	Mean (SD) prior knowledge/experience of autism (max = 7)
Child A	Told AUT + info	20	43.70 (13.87)	10:10	3.70 (1.30)
	Told AUT + no info	20	43.80 (15.54)	10:10	4.05 (1.67)
	Not told AUT + no info	20	44.95 (14.52)	10:10	3.40 (1.50)
Child B	Told AUT + info	20	43.50 (14.89)	10:10	3.60 (1.19)
	Told AUT + no info	20	43.80 (14.71)	10:10	3.10 (1.37)
	Not told AUT + no info	20	43.53 (14.79)	10:10	3.25 (1.37)

## Materials

### Witness Videos

As part of a larger study on the eyewitness capabilities of child witnesses with and without an autism diagnosis (see Henry et al. 2017a), 18 children on the autism spectrum received a ‘Best-Practice’ police interview, conducted in line with Achieving Best Evidence (Home Office 2011) guidelines. From this sample, we selected two videos to show to a sample of mock jurors. These videos were selected as they were of good audio and visual quality, and because their parents had provided consent for the videos to be used in a juror perception study (note: no other videos from the larger study were available for this purpose).

The two videos each depicted boys sitting alongside the same interviewer (a postdoctoral research associate who had completed a 1-week Investigative Interviewing Victim and Witnesses Training Course provided by the UK’s Metropolitan Police Service). 1 week prior to the interview, both boys had watched a video of a staged event involving two men giving a talk about what school was like a long time ago. The talk had educational content but also involved a minor mock crime (the theft of a phone). As well as receiving an immediate, brief interview about what they saw (which took place on the same day; see Henry et al. 2017b, for details), the children took part in a full investigative interview approximately 1 week later conducted according to best-practice police guidelines currently used in England and Wales (see Henry et al. 2017a). This latter full investigative interview was shown to the mock jurors as part of the current study (as would happen in a real case). It comprised three distinct phases.

The first phase comprised ‘rapport building’, in which the interviewer asked the child some neutral questions not related to the staged event, designed to elicit positive answers and set a good tone for the subsequent interview. For Child A, this involved a conversation about what the child did on the weekend (his birthday party); and for Child B, a conversation about what the child had for lunch, and what he was planning to do after school on that day (going to the park). Phase two comprised a ‘truth and lies exercise’, in which the interviewer used a scenario to determine whether the child could tell the difference between truth and lies (e.g., the interviewer pointed to a blue door and said to the child: ‘That door is bright red. Is that the truth or a lie?’). Both children were able to respond accurately to the truth and lies question (they confirmed that the interviewer had told a lie and provided the correct answer). The interviewer concluded this portion of the interview by explaining to the child that it was important that they tell the truth and not make anything up, and that it was fine to say that they did not know the answer to a question. Phase three comprised the ‘investigative interview’, in which the interviewer questioned the child about what they saw using Achieving Best Evidence (Home Office 2011) principles; guidelines used for investigative interviewing in England and Wales. Specifically, the interviewer elicited a free recall account from the child by stating: ‘Tell me what you remember about what you saw’. The interviewer then asked open questions based upon what the child had said in their free recall. Given that the nature and content of the questioning depended on the information the child provided during their free recall, ‘prompts’ from the interviewer were individually tailored for each child, but both children were prompted a similar number of times (Child A = 16 times; Child B = 18 times).^3^

### Interview Performance of Each Child Witness

In the investigative interview, both children recalled key aspects of the staged event: (1) that it involved two men, who they could briefly describe; (2) that it involved content related to Victorian times; and (3) that there was an issue regarding a phone. Child A recalled the event in somewhat more detail than Child B. For example, whilst Child B simply stated that “one [man] left their phone behind”, Child A recalled significantly more about the phone (“one man said ‘make sure no one takes my phone’ then the other man took his phone… he put his phone on the chair and not the floor… and he took it thinking it was his phone”). Indeed, calculating the total amount of details recalled (as per Henry et al. 2017a, b), Child A recalled 43 details, whilst Child B recalled 27 details. Importantly, these figures represent the total amount of information the children provided, irrespective of its accuracy. This is because jurors do not know whether information recalled is correct or incorrect [Accuracy rates were: Child A (76.74%) and Child B (85.18%)—with both showing fairly high percentage accuracy rates]. Child A’s interview lasted 10 min 16 s (3 min 20 s for rapport building, 30 s for truth and lies, 6 min 26 s for the investigative interview) and Child B’s interview lasted 6 min 44 s (1 min 13 s for rapport building, 42 s for truth and lies, 4 min 49 s for the investigative interview).

#### Cognitive Characteristics of Each Child Witness

The children were both boys, of a similar age. They were also of similar (average) cognitive ability (as measured using the Wechsler Abbreviated Scale of Intelligence, WASI-II, Wechsler and Zhou 2011) and receptive language ability (as measured using the British Picture Vocabulary Scale, BPVS-III, Dunn et al. 2009). Child A, however, had higher scores on some expressive language measures: subtests of the Expressive Language Test (ELT-2, Bowers et al. 2010) and the Clinical Evaluation of Language Fundamentals (CELF-4, Semel et al. 2006). The characteristics of the children are presented in Table 2.

**Table 2:** Characteristics of the two child witnesses

	Child A	Child B
Age	9 years 11 months	10 years 9 months
WASI-II verbal IQ^a^	53	56
WASI-II non-verbal IQ^a^	46	43
WASI-II full-scale IQ^2^	99	99
BPVS-III^b^	92	89
ELT-2 sequencing^b^	113	114
ELT-2 grammar and syntax^b^	111	92
CELF-4 recalling sentences^c^	11	9
CELF-4 formulated sentences^c^	12	5

^a^T-scores (mean 50, SD 10)

^b^Standardised scores (mean 100, SD 15)

^c^Scaled scores (mean 10, SD 3)

#### Behavioural Characteristics of Each Child Witness

To further explore the characteristics of the children in the videos, we asked 24 lay people to rate the videos on a series of behavioural characteristics, all on a series of 7-point Likert scales (from 1 = not at all, to 7 = very much). As can be seen in Table 3, this revealed that Child A was rated as significantly more monotonous than the Child B, while Child B was rated as significantly less composed, coherent, and focused than Child A, and also as showing significantly less appropriate use of vocabulary.

**Table 3:** Lay person ratings of behavioural characteristics for the child witnesses

	Child A	Child B	Differences
How repetitive the child’s account was	3.70 (1.74)	2.96 (1.52)	*t*(46) = 1.61, *p* = .11
How monotonous the child’s tone of voice was	5.39 (1.53)	3.29 (1.33)	*t*(46) = 4.94, *p* < .001
How composed the child appeared	5.48 (1.24)	3.29 (1.30)	*t*(46) = 5.96, *p* < .001
How appropriate the vocabulary the child used was	5.61 (1.16)	4.79 (1.35)	*t*(46) = 2.19, *p* = .03
How coherent the child’s speech was	5.09 (1.28)	4.08 (1.41)	*t*(46) = 2.60, *p* = .01
How clear the child’s speech was	4.57 (1.53)	4.83 (1.37)	*t*(46) = − 0.50, *p* = .62
How natural the child’s facial expression was	5.09 (1.47)	4.75 (1.42)	*t*(46) = 0.81, *p* = .42
How natural the child’s body language was	4.87 (1.58)	4.17 (1.68)	*t*(46) = 1.43, *p* = .16
How natural the child’s eye contact was	4.61 (1.37)	4.17 (1.43)	*t*(46) = 1.14, *p* = .26
How natural the child’s gestures were	4.65 (1.56)	3.83 (1.46)	*t*(46) = 1.93, *p* = .06
How focused the child was	5.43 (1.47)	3.46 (1.32)	*t*(46) = 4.85, *p* < .001

### Procedure for Mock Jurors

Prior to viewing the videos, the mock jurors in the ‘Told AUT + info’ condition were informed that the child had an autism diagnosis and were also asked to read some information detailing the key features and characteristics of the condition (see “Appendix”); mock jurors in the ‘Told AUT + no info’ condition were informed that the child in the video had an autism diagnosis, but were not given any additional information about autism; and mock jurors in the ‘Not told AUT + no info’ condition were not given any information about the child’s diagnosis. It was explained to the jurors that the video that they were about to view comprised three distinct phases: rapport, truth and lies and the main investigative interview.

After watching one of the videos (of Child A or Child B), participants completed a pen and paper credibility questionnaire, which asked them to rate the following aspects of the witness’ credibility on 11 7-point Likert scales (from 1 = not at all, to 7 = very much): accuracy; convincingness; confidence in what was said; confidence in demeanour; competence; honesty; believability; completeness of account; level of cognitive functioning; capability to testify; and overall performance (adapted from Henry et al. 2011; Peled et al. 2004).

At the end of the study, after participants had been debriefed about the aims of the research, those who were not informed of the child’s diagnosis were asked whether they had guessed the child in the video was diagnosed with autism. For Child A, none of the 20 mock jurors in the ‘Not told AUT + no info’ condition guessed that the child was on the autism spectrum, and for Child B, three of the 20 jurors guessed the child’s diagnosis. There was no significant difference in the likelihood of the jurors guessing the child’s diagnosis as a function of whether they viewed Child A or Child B’s video, χ^2^(1) = 0.07, *p* = .23. Participants were also asked how much knowledge and experience they had of autism, prior to taking part in this research, rated on a 7-point Likert scale from 1 (no knowledge/experience) to 7 (very knowledgeable/experienced) (see Table 1). One way analyses of variance (ANOVAs) revealed no significant differences in mock jurors’ knowledge/experience of autism as a function of Condition (‘Told AUT + info’, ‘Told AUT + no info’, ‘Not told AUT + no info’) either for those who viewed the video of Child A, *F*(2, 57) = 0.94, *p* = .39, *η*_p_^2^ = 0.03, or Child B, *F*(2, 57) = 0.76, *p* = .47, *η*_p_^2^ = 0.03. Further, there was no significant difference in knowledge/experience of autism between those who viewed the video of Child A (mean = 3.72, SD = 1.50) or Child B (mean = 3.32, SD = 1.31), *F*(1, 118) = 2.43, *p* = .12, *η*_p_^2^ = 0.02.

Ethical approval was obtained from the Research Ethics Committee at the university at which the research was conducted.

## Results

Mean scores for the 11 credibility questions answered by mock jurors (in each of the three conditions) in relation to the video that they watched (Child A or Child B) are given in Table 4.

**Table 4:** Mean overall credibility ratings (on a 7-point Likert scale) for the children across the three conditions (*Told AUT* + *info, Told AUT* + *no info*, and *Not told AUT* + *no info*) (n = 120)

	Child A			Child B		
	Told AUT + info	Told AUT + no info	Not told AUT + no info	Told AUT + info	Told AUT + no info	Not told AUT + no info
	n = 20	n = 20	n = 20	n = 20	n = 20	n = 20
Mean credibility score (across all 11 items)	4.45 (1.01)	5.10 (0.78)	4.51 (1.20)	4.55 (0.92)	3.86 (0.90)	3.68 (1.17)
How *convincing* was the child?	4.60 (1.43)	5.25 (0.85)	4.70 (1.62)	4.45 (1.10)	3.75 (1.21)	3.75 (1.25)
How *confident* was the child *in what they said*?	4.45 (1.47)	5.05 (1.19)	4.05 (1.57)	4.45 (1.15)	3.80 (1.28)	3.70 (1.52)
How *confident* was the child *in their demeanour*?	3.90 (1.02)	4.25 (1.07)	3.45 (1.54)	4.20 (1.28)	3.65 (1.27)	3.45 (1.43)
How *competent* was the child?	4.35 (0.87)	5.05 (1.19)	4.35 (1.09)	4.45 (0.94)	3.90 (1.07)	3.55 (1.28)
How *honest* was the child?	5.20 (1.24)	6.10 (1.02)	5.50 (1.15)	5.75 (1.02)	5.05 (1.19)	4.45 (1.19)
How *believable* was the child?	4.95 (1.32)	5.50 (0.89)	4.90 (1.48)	4.70 (0.92)	4.10 (1.21)	3.95 (1.47)
How *complete* was the child’s account?	4.10 (1.74)	4.50 (1.28)	4.00 (1.49)	3.75 (0.97)	3.00 (0.79)	3.20 (1.44)
What was the child’s *overall level of cognitive functioning*?	4.60 (1.54)	4.90 (1.25)	4.70 (1.49)	4.40 (1.09)	3.80 (0.83)	3.75 (1.48)
What was the child’s *capability to testify*?	4.30 (1.52)	5.15 (1.31)	4.60 (1.63)	4.45 (1.32)	3.70 (1.30)	3.50 (1.39)
How *good was this child overall*?	4.15 (1.50)	5.20 (1.20)	4.60 (1.50)	4.50 (1.28)	3.65 (1.27)	3.50 (1.36)

Prior to exploring the effect of providing jurors with information about autism and/or the child’s diagnostic label, a principle component analysis (PCA) using Oblimin rotation was conducted on the 11 credibility ratings (to reduce the dimensionality and noise in the data). The Kaiser-Mayer-Olkin measure verified the sampling adequacy for the analysis, KMO = 0.92, and KMO values for all individual items were > 0.8; well above the acceptable limit of 0.5 (Field et al. 2010). Bartlett’s test of sphericity, χ^2^ (55) = 1157.28, *p* < .001, indicated that correlations between items were sufficiently large for PCA. An initial analysis was run to obtain eigenvalues for each component in the data. One component had an eigenvalue over Kaiser’s criterion of 1 and explained 67.2% of the variance. The scree plot showed an inflection that would justify retaining component 1 (see Table 5 for factor loadings). All 11 items clustered on the same component, suggesting that component 1 represented ‘overall credibility’. This component was used as a dependent variable for subsequent analyses.^4^

**Table 5:** Principal components analysis loadings (n = 120)

	Factor loadings
	Overall credibility
What was the child’s capability to testify?	0.88
How good was the child overall?	0.88
What was the child’s overall level of cognitive function?	0.85
How accurate was the child?	0.84
How believable was the child?	0.84
How convincing was the child?	0.84
How competent was the child?	0.83
How complete was the child’s account?	0.82
How confident was the child in what they said?	0.80
How honest was the child?	0.75
How confident was the child in their demeanour?	0.65

To explore mock jurors’ perceptions of the witness’ credibility, two one-way between participants ANOVAs were used (one for each child) with Condition (‘Told AUT + info’, ‘Told AUT + no info’, ‘Not told AUT + no info’) as the independent variable and the ‘overall credibility’ component (extracted from the PCA) as the dependent variable. There was no significant effect of Condition for Child A, *F*(2, 57) = 2.55, *p* = .09, *η*_p_^2^ = 0.08, although Condition was significant for Child B, *F*(2, 57) = 4.18, *p* = .02, *η*_p_^2^ = 0.13. Bonferroni corrected post-hoc *t* tests revealed that Child B was judged as more credible in ‘Told AUT + info’ condition relative to the ‘Not told AUT + no info’ condition (*p* = .02), with all other comparisons between conditions non-significant (*p*s > .05).

Next, bivariate correlations were used to explore the relationship between mock jurors’ credibility ratings and their knowledge/experience of autism. These revealed that there was a significant positive relationship between scores on the ‘overall credibility’ component (extracted from the PCA) and mock jurors’ ratings of prior knowledge/experience of autism for those who viewed the video of Child B (*r* = .34, *p* < .05), but not for those who viewed the video of Child A (*r* = − .01, *p* = .93).

## Discussion

This preliminary study examined how mock jurors perceived the credibility of two child witnesses on the autism spectrum and examined whether informing mock jurors about the child’s autism diagnosis (with or without the provision of additional information about autism) affected mock jurors’ credibility ratings. Results demonstrated that mock jurors’ credibility ratings were, to an extent (i.e., for only one child witness), influenced by the provision of the child’s diagnostic label and information about autism. Specifically, ratings of credibility were lower for Child B if no autism label or autism information had been provided, relative to both being provided. However, this was not the case for Child A (credibility ratings did not vary with the provision of information about autism or the child’s diagnostic label). It is important to consider why the results might differ for these two child witnesses on the autism spectrum.

One potential reason relates to the level of detail provided by each child during their investigative interview. Previous juror perception studies (e.g., Henry et al. 2011) have shown that witnesses who recall more information about an event are perceived to be more credible. Consistent with this suggestion, Child A (who was judged to be more credible on many items rated by mock jurors) recalled more details at interview (43) than Child B (27). Hence, there may have been less need to use Kelley’s (1971, 1972) ‘discounting principle’ (that one explanation is diminished when observers are alerted to an alternative one) with Child A because his account was fuller and, possibly for this reason, more convincing. Importantly, for witnesses diagnosed with autism, a growing body of research has demonstrated that autistic children often recall less information than their non-autistic peers, largely during free recall (e.g., Bruck et al. 2007; Henry et al. 2017b; Mattison et al. 2015; McCrory et al. 2007). Whilst this is not always apparent during more structured questioning (e.g., Henry et al. 2017a), there is still significant variability in the amount that children (including those on the autism spectrum) recall: some individuals provide detailed and extensive accounts, whereas others provide sparser reports, even with more structured questioning (cf. Henry et al. 2017a). It is particularly important for jurors to be made aware of this prior to evaluating an autistic child’s testimony, since media representations of people on the autism spectrum commonly emphasise prodigious memory skills, potentially raising expectations of autistic people’s abilities to an unrealistically high level (Draaisma 2009).

The two children in the videos may also have differed in other important ways that had a bearing on mock juror credibility ratings. Whilst similar in age, gender, IQ, and receptive language, the two children may have differed in their manifestation of autism (i.e., the way in which their behaviours appeared to mock jurors). A key feature of autism is that different individuals display different behaviours and to different degrees (as emphasised in the information given to jurors in the ‘Told AUT + info’ condition); thus, it was not surprising that the two autistic children who featured in this study varied in their behaviour and demeanour. Our sample of 24 lay people who rated the videos on a series of behavioural characteristics highlighted several differences. While Child A was rated as significantly more monotonous than the Child B, Child B was rated as significantly less composed, coherent, and focused than Child A, whilst also being rated as showing significantly less appropriate use of vocabulary (the latter observation being in line with the poorer expressive language scores for Child B relative to Child A, on standardised tests). Again, Kelley’s (1971, 1972) discounting principle may only have applied to Child B, who displayed atypical behavioural characteristics possibly associated with autism (i.e., being less focused, composed, coherent). This might link to our mock jurors’ ratings of their knowledge/experience of autism, which correlated with credibility ratings for Child B but not Child A. These findings suggest that knowledge/experience of autism may affect credibility judgements only when the child is displaying more atypical behavioural characteristics, which has implications for children who may not match the stereotypical view of an autistic child, such as some girls on the autism spectrum (Gould and Ashton-Smith 2011), an important area that warrants further investigation.

Overall, the results of this preliminary study highlight how care must be exerted regarding how information about autism is presented to juries. We suggest that, rather than providing generic information about autism, information specific to each autistic child should be presented; explicitly outlining how their autism manifests and how it might impact on their testimony (if at all). Surveys have highlighted how legal professionals (e.g., police officers, barristers), even with fairly good knowledge of the key characteristics of autism, often lack confidence in their abilities to make accommodations and adjustments to meet the needs of autistic witnesses and defendants (George et al. 2018; Maras et al. 2017). As such, they may be unsure how to present information about autism to a jury. In England and Wales, the Registered Intermediary (RI) role has been introduced; initially as a pilot scheme (in 2004) before being rolled out more widely in 2009 (Cooper 2016; Cooper and Wurtzel 2014; Plotnikoff and Woolfson 2015). RIs are impartial, trained professionals who facilitate communication between vulnerable witnesses^5^ and criminal justice professionals. RIs are individually ‘matched’ to vulnerable witnesses based on the expertise of the RI and the needs of the witness (as such, an autistic child will likely be matched to an RI with expertise in both autism and working with child witnesses). As part of their wide-ranging role, the RI will conduct detailed assessments of the vulnerable witness, to identify how to facilitate fair and appropriate access to justice for that specific individual. An RI would, therefore, be in an excellent position to prepare individualised information about a witness for a jury, which the current research suggests is urgently needed. There is growing international interest in implementing RI schemes outside of England and Wales (Cooper and Mattison 2017; Plotnikoff and Woolfson 2015), with schemes being successfully implemented in other jurisdictions (Cooper 2016; Cooper and Wurtzel 2014). Until such a procedure exists in other countries, expert witnesses (or similar) may be able to provide such information to juries, in collaboration with the vulnerable individual (if possible) and those who know them well (e.g., family members, teachers, support workers).

Finally, it is important to highlight the strengths and limitations of this research. Most juror perception research uses transcripts of evidential interviews (e.g., Henry et al. 2011), enabling a high degree of experimental control but lacking real-world applicability. The current study presented ecologically valid video-recorded interviews, as would be presented to jurors in a real case. Yet this does come at a cost—whilst the results showed how credibility ratings differed for each child depending on the information provided to jurors, it was unclear exactly why these differences occurred. Within the constraints of this preliminary study, it was not possible to provide an objective measure of the children’s levels of autistic traits, nor explore precisely which aspects of the children’s testimony might have affected credibility ratings (e.g., by interviewing mock jurors about their rationale for their credibility judgements). It is important that future research (using a wider range of child witnesses) examines this more systematically.

Further, the videos shown to mock jurors were taken from an experimental study in which the children had viewed a video of a mild mock theft (as observers, rather than as active participants). In real-life, undertaking an investigative interview with a police officer (potentially about a serious or emotionally upsetting event) could cause high levels of anxiety and stress for a witness. This may be especially true of children on the autism spectrum, with one review suggesting that between 11 and 84% of autistic children experience impairing anxiety to some degree (White et al. 2009). This could affect how the child presents during interview, which may affect juror perceptions of credibility. Future work should, if possible, explore how jurors rate the credibility of autistic child witnesses in real-life evidential interviews.

## Conclusion

This preliminary study demonstrated that providing mock jurors with a child’s diagnostic label and informing them of generic information about autism affected credibility ratings, but not for both of the autistic children included. We tentatively suggest that differences occurred in credibility ratings for one child and not the other due to factors such as the amount of information recalled or the overtness of the children’s behavioural atypicalities. Hence, caution should be exercised when providing jurors with information about a child’s diagnostic label and autism more generally, and any information provided to jurors should be tailored to the specific profile of the child. It is crucial for criminal justice professionals to consider—on an individual basis—how best to present information about child witnesses to a jury, especially in relation to those on the autism spectrum. It will also be important for future work to include adult witnesses on the autism spectrum, to explore the impact of age on judgements of witness credibility.

## Appendix: Instructions Provided to Participants in the *AUT* + *info* Condition

### What is Autism?

Autism is a developmental condition that affects how a person communicates with, and relates to, other people. For example, children with autism may not understand the unwritten social rules that children without the condition inherently pick up on.

Some of the behavioural features of autism often include:

- Idiosyncratic speech and odd intonations: For example, a child with autism’s speech might be particularly flat or ‘monotone’. It may also be high-pitched, or have unusual rhythm and loudness.
- Literality: Children with autism can be very literal in what they say and can have difficulty understanding jokes, metaphor and sarcasm. For example, “that’s cool” might be taken to mean that it is cold.
- Facial expressions and gestures: Children with autism may use unusual, or a limited range of, facial expressions. They can find it difficult to use expressive gestures appropriately and to convey the meaning of what they are saying.
- Topics of conversation: Children with autism sometimes go off-topic in their story telling, and find it difficult to tell their story according to the listener’s needs.
- Repetitive, nervous and ‘stimmming’ behaviours: Children with autism often show unusual movements, which might include rocking, hand flapping, finger flicking, twitchy and repetitive movements.
- Inappropriate eye contact: Children with autism sometimes make unusual eye contact, or avoid making eye contact altogether.

Importantly, autism is a spectrum condition. This means that, while people with autism share certain difficulties, they are affected by it in different ways and not all children with autism will display the behaviours just described, or to the same degree. It is often referred to as a ‘hidden’ disability, because it is not always obvious that a person has an autism diagnosis.

## Footnotes

1 There is debate regarding the way autism is—and should be—described. In this article, we use both identity-first language (i.e., autistic children) as well as person-first language (i.e., children with autism) to respect this diversity of views (see Kenny et al. 2016).

2 In England and Wales, the term ‘expert witness’ refers to a witness who is qualified to provide a statement of opinion to a court on a matter requiring expertise.

3 These prompts comprised a mixture of ‘reiterations’ (e.g., “you said they got their maths wrong…”; five such prompts for Child A and nine for Child B), ‘requests for additional information’ (e.g., “tell me more about that”; five such requests for Child A and seven for Child B), and ‘clarifications’ (e.g., Child: ‘the shoe looks like this [points], Interviewer: ‘ok –like black trainers?’; six such requests for Child A and two for Child B).

4 Note that all analyses were also run with the mean score across all 11 credibility items (opposed to the ‘overall credibility’ component derived from the PCA) and the results remained the same.

5 The term ‘vulnerable witness’ includes anyone under the age of 18 years, as well as children or adults with an autism diagnosis (although those who fall under the category of vulnerable witness is much broader than this, including victims of sexual offences and other serious crimes, victims who are persistently targeted, and victims with communication difficulties, among others).
